# Sex-Dependent Variation of Pumpkin (*Cucurbita maxima* cv. Big Max) Nectar and Nectaries as Determined by Proteomics and Metabolomics

**DOI:** 10.3389/fpls.2018.00860

**Published:** 2018-06-29

**Authors:** Elizabeth C. Chatt, Patrick von Aderkas, Clay J. Carter, Derek Smith, Monica Elliott, Basil J. Nikolau

**Affiliations:** ^1^Department of Biochemistry Biophysics and Molecular Biology, Iowa State University, Ames, IA, United States; ^2^Department of Biology, University of Victoria, Victoria, BC, Canada; ^3^Department of Plant and Microbial Biology, University of Minnesota Twin Cities, St. Paul, MN, United States; ^4^UVic Genome BC Protein Centre, Victoria, BC, Canada

**Keywords:** metabolomics, proteomics, nectar, *Cucurbita*, pumpkin, floral sex

## Abstract

Nectar is a floral reward that sustains mutualisms with pollinators, which in turn, improves fruit set. While it is known that nectar is a chemically complex solution, extensive identification and quantification of this complexity has been lacking. *Cucurbita maxima* cv. Big Max, like many cucurbits, is monoecious with separate male and female flowers. Attraction of bees to the flowers through the reward of nectar is essential for reproductive success in this economically valuable crop. In this study, the sex-dependent variation in composition of male and female nectar and the nectaries were defined using a combination of GC-MS based metabolomics and LC-MS/MS based proteomics. Metabolomics analysis of nectar detected 88 metabolites, of which 40 were positively identified, and includes sugars, sugar alcohols, aromatics, diols, organic acids, and amino acids. There are differences in 29 metabolites between male and female nectar. The nectar proteome consists of 45 proteins, of which 70% overlap between nectar types. Only two proteins are unique to female nectar, and 10 are specific to male nectar. The nectary proteome data, accessible at ProteomeXchange with identifier PXD009810, contained 339 identifiable proteins, 71% of which were descriptively annotatable by homology to Plantae. The abundance of 45 proteins differs significantly between male and female nectaries, as determined by iTRAQ labeling. This rich dataset significantly expands the known complexity of nectar composition, supports the hypothesis of H+-driven nectar solute export, and provides genetic and chemical targets to understand plant–pollinator interactions.

## Introduction

Nectar is the most common floral reward used by angiosperms to mediate a mutualistic relationship with pollinators, and improves the plant’s reproductive success by promoting outcrossing (Mitchell et al., 2009). In crops such as oilseed rape (Carruthers et al., 2017), sunflower (Mallinger and Prasifka, 2017), and pumpkin (Nepi and Pacini, 1993), variations in nectar composition and volume directly influence the frequency of pollinator visitation. Because 87 out of 115 global food crops are dependent on or achieve improved fruit set through animal-mediated pollination (Klein et al., 2007), a potential future breeding goal could target improved nectar traits. However, in order to exploit this trait, a more comprehensive understanding of nectar composition is needed.

Nectar is a complex solution that, depending on the species, may contain some or all of the following constituents: carbohydrates, amino acids, vitamins, alkaloids, phenolics, terpenoids, lipids, metal ions, hormones, and proteins (Richardson et al., 2015; Roy et al., 2017). The two most predominant classes of metabolites are carbohydrates followed by amino acids (Lüttge, 1977). A system of nectar classification based on the ratios of predominant sugars proposed by Baker and Baker (1983) defines four classes of nectar: hexose-dominant, hexose-rich, sucrose-dominant, and sucrose-rich. Different clades of animals are attracted to different hexose-sucrose ratios and nectar amino acid profiles (Baker and Baker, 1983; Gardener and Gillman, 2002; Hendriksma et al., 2014; Nepi, 2014). Thus, nectar ecology studies typically define nectar composition based upon targeted analyses of predominant sugars and occasionally the amino acids. To date, few studies have applied metabolomics techniques to study nectar composition (Kram et al., 2008; Bender et al., 2012, 2013; Noutsos et al., 2015). Metabolomics, as used in this study, can potentially detect novel secondary metabolites important for pollinator attraction and health, which are instrumental in sustaining the ecosystem service of pollination.

While most analyses have concentrated on small molecular weight compounds, such as sugars, recent studies have revealed an abundant and diverse proteome. Nectar proteins (nectarins) studied thus far either display anti-microbial properties (Carter and Thornburg, 2004; Kram et al., 2008; Hillwig et al., 2010, 2011; Zhou et al., 2016) or modify carbohydrates (González-Teuber et al., 2010; Nepi et al., 2011a, 2012). A nectar redox cycle discovered in *Nicotiana* nectar is based on anti-microbial nectarins that produce hydrogen peroxide, which inhibits microbial infection of the nectary (Carter and Thornburg, 2000, 2004; Carter et al., 2007). On occasion, the microbial defense function and carbohydrate modification reactions overlap. For example, in *Cucurbita pepo* nectar the degradation of pathogen elicitor xylans by β-xylosidases can suppress pathogen infection (Nepi et al., 2011a, 2012).

*Cucurbita maxima* cv. Big Max is an ideal system to study sex-dependent variations of nectar, because it is a monoecious plant with unisexual flowers. Male flowers of *C. maxima* produce three times less nectar than females and out-number the female flowers 3:1 (Ashworth and Galetto, 2002). In both the male and female flowers, nectariferous tissue lines the adaxial receptacle surface. Secretion of sucrose-dominant nectar produced by starch hydrolysis begins at dawn the day of anthesis and ceases by noon at which point reabsorption of unconsumed nectar occurs (Ashworth and Galetto, 2002). Detailed studies of nectar dynamics in *C. pepo* have found significant sex-dependent variation when comparing the nectar sugar concentration, nectar volume, and rates of nectar production (Nepi et al., 2001).

The main objective of this study was to determine whether sex-dependent variation occurs in nectar composition at the level of both the metabolome and proteome, and secondarily to define potential metabolic links between the proteomes and the production of nectar metabolites. Thus, the combined application of metabolomics and proteomics analyses better define nectar biology of *Cucurbita maxima* cv. Big Max. The nectar of male and female flowers was analyzed using a GC-MS based untargeted metabolomics approach, as well as targeted amino acid profiling. For the first time in cucurbits, the proteomes were examined using LC-MS and iTRAQ (isobaric tag relative and absolute quantitation) to measure nectary protein expression. The collected omics-data were interpreted in the context of two models of nectar secretion, the merocrine and eccrine models (Roy et al., 2017).

## Materials and Methods

### Plant Materials, Growth Conditions, Sample Collection

Seeds of *Cucurbita maxima* cv. Big Max were sown in 4-inch peat pots in a greenhouse. Approximately 2 weeks later, 17 seedlings that were at the two-leaf developmental stage were transplanted to a field plot located at the North Central Regional Plant Introduction Station, Ames, IA, United States (42°00′40.8″N 93°39′46.9″W). Plants were enclosed by a 4.5 m × 12 m × 2 m polyethylene (natural amber) mesh cage to reduce accessibility by insects and the consumption of nectar by pollinators. All nectar and nectary samples were collected at anthesis between 8:00 am and 11:00 am. Flowers were removed from the plant before collecting nectar using an AlphαPette^TM^ pipette with sterile tips. Nectary tissue was then dissected from the flower using a sterile scalpel. Nitrile gloves were worn during all collections. All samples were immediately flash-frozen and stored in liquid nitrogen before long term storage at −80°C.

### Nectar Metabolite Extraction and Analysis

#### Untargeted Metabolomics

An untargeted metabolomics extraction method was adapted from Schmidt et al. (2011). Each extraction used 20 μL of nectar collected from a single flower. For biological replication purposes, extracts were prepared from at least six independent male and female flowers, and they were processed and analyzed individually without pooling. Prior to the extraction, internal standards (5 μg nonadecanoic acid and 2 μg ribitol) were added to the nectar sample. The mixture was immediately incubated for 10 min with 3.5 mL of hot methanol (60°C) followed by sonication for 10 min. Chloroform (3.5 mL) and water (3 mL) were added and the mixture was vortexed after the addition of each solvent. The mixture was centrifuged, and the top polar, and bottom non-polar layers were recovered separately. The entire non-polar layer (3 mL) and 2 mL of the polar layer were transferred to individual 2 mL screw-cap glass vials and dried overnight by lyophilization. The analysis of predominant sugars (glucose, fructose, and sucrose) was conducted with a 1-μL sample of nectar, which was spiked with 25 μg ribitol and the mixture was dried overnight by lyophilization. The dried polar extracts and the predominant sugar preparations underwent methoximation for 90 min with 20 mg mL^−1^ methoxyamine hydrochloride in pyridine at 30°C with continuous agitation. All samples including the dried non-polar extracts were silylated for 30 min at 60°C with BSTFA/TMCS (*N*,*O*-Bis(trimethylsilyl)trifluoroacetamide/Trimethylchlorosilane). The predominant sugar samples were diluted with 1 mL pyridine. Samples were analyzed using a GC/GC-MS consisting of an Agilent Technologies Model 6890 gas chromatograph equipped with an Agilent HP-5ms Inert (30 m, 0.25 mm, 0.25 μm) column and a low thermal mass (LTM) oven, which was coupled to Model 5975C mass spectrometer. GC was conducted with a helium gas flow rate of 1 mL min^−1^, 1 μL injection, and a temperature gradient of 80°–320°C at a rate of 5°C min^−1^. A heart-cut method, which diverted gas flow to a secondary LTM column at the elution times for fructose, glucose, and sucrose, was utilized to analyze the minor components of the polar extracts. Deconvolution and integration of resulting spectra was performed with AMDIS (Automated Mass Spectral Deconvolution and Identification System) software. Analyte peaks were identified by comparing mass spectra and retention indices to the NIST14 Mass Spectral Library and when possible, to authentic standards to confirm chemical identification (Stein, 1999).

#### Targeted Amino Acid Analysis

Analysis of amino acids was performed using the Phenomenex EZ:Faast^TM^ kit for free amino acids (Torrance, CA, United States). Each sample (60 μL nectar per extraction) consisted of nectar pooled from four individual flowers. Six replications were analyzed for each sex. Sample preparation from solid phase extraction to derivatization were completed according to the manufacturer with one adjustment: after addition of the norvaline internal standard to each sample, 125 μL of 10% propanol/20 mM HCl was added to acidify the sample. Following derivatization, samples were concentrated under a stream of nitrogen gas before amino acids were analyzed using an Agilent Technologies model 6890 gas chromatograph coupled to a model 5973 mass selective detector capable of electrical ionization (EI). The GC-MS instrument settings followed the manufacturer’s recommendations.

### Nectar Proteomics

Nectar samples were collected from three individual flowers of both male and female flowers, and these samples were pooled to average biological differences among the two flower types. These pooled nectar samples were analyzed individually for both male and female flowers. Nectar samples were first reduced with dithiothreitol for 30 min at 37°C and alkylated with iodoacetamide for 30 min at 37°C. Each sample was digested with 2 μg trypsin for 16 h at 37°C). Desalting was completed using a Waters HLB Oasis column followed by concentration in a Speed-Vac. Peptide mixtures were rehydrated to 50 μL using a solution of 2% acetonitrile and 2% formic acid. Six microliters were injected for LC-MS/MS analysis using a Thermo Scientific EASY-nLC II system coupled to an LTQ Orbitrap Velos Pro mass spectrometer equipped with a Nanospray Flex source. The LC system utilized a Magic C-18AQ reversed-phase pre-column (100 μm I.D., 2 cm length, 5 μm, 100 Å) and in-house prepared reversed-phase nano-analytical column packed with Magic C-18AQ (75 μm I.D., 15 cm length, 5 μm, 100 Å). The solvent system consisted of buffers A (2% acetonitrile, 0.1% formic acid) and B (90% acetonitrile, 0.1% formic acid) with a 90 min linear gradient (0 min: 5%B; 90 min: 30%B; 2 min: 100%B; 8 min: 100%B) at a flow rate of 300 nL min^−1^. Orbitrap nano-electrospray ion source was set to a voltage of 2.5 kV and capillary temperature of 250°C. The scan m/z range was 400–2000. The ten most intense ions (charge state 2–4 exceeding 50,000 counts) were selected for ion trap collision induced dissociation (CID) and detection in centroid mode. Common human keratin and porcine trypsin peptide masses were excluded from MSMS selection during the analysis.

### Nectary Proteomics

#### Protein Extraction and iTRAQ Labeling

Each biological replicate consisted of nectary tissue from a single flower with a total of two female replicates and five male replicates. To extract proteins, nectaries were pulverized under liquid nitrogen and solubilized in 4 M urea/0.1 M triethylammonium bicarbonate (TEAB). Proteins were precipitated overnight in acetone and dissolved in 4 M urea/0.1 M TEAB.

Protein concentrations were determined using a bicinchoninic acid (BCA) protein assay. Ten volumes of acetone at −20°C were used to precipitate 100 μg of extracted protein overnight. The resulting protein pellet was dissolved in 0.5 M TEAB/0.2% sodium dodecyl sulfate for 4 h at 4°C before reduction with 50 mM tris (2-carboxyethyl) phosphine hydrochloride (TCEP) for 1 h at 60°C. Alkylation with 200 mM methyl methanethiosulfonate (MMTS) at room temperature for 10 min was completed prior to overnight in-solution digestion at 37°C with 10 μg trypsin prepared in 100 mM TEAB. Digests were dried in a Speed-Vac before rehydration with 30 μL of 0.5M TEAB/50 μl isopropanol. iTRAQ labels were added to each sample before being pooled and concentrated to a final volume of approximately 100 μL using a Speed-Vac.

#### Chromatography and Mass Spectrometry

The iTRAQ labeled peptide sample was fractioned and concatenated using an Agilent 1290 HPLC with a Waters XBridge C18 column (250 mm × 4.6 mm, 5 μm, 300 Å) and solvent system consisting of buffers A (10 mM ammonium hydroxide, pH10) and B (80% acetonitrile, 10 mM ammonium hydroxide, pH 10). The column was equilibrated in buffer A at a flow rate of 0.75 mL min^−1^ before a gradient of 5–45% buffer B was applied over 75 min. Fractions were collected every minute for 96 min, concentrated by lyophilization, and concatenated into 24 fractions by combining every 24th fraction. Fractions were de-salted using C18 StageTips and rehydrated with 20 μL of 2% acetonitrile/3% formic acid. For LC-MS/MS peptide sequencing, 5 μL aliquots of each fraction were injected into a Thermo Scientific EASY-nLC II system coupled to an LTQ Orbitrap Velos Pro mass spectrometer equipped with a Nanospray Flex source. The same columns, solvent system, and mass spectrometer parameters as described for nectar peptide sequencing in the previous section were used with the following adjustments. Peptides were separated using a 120 min gradient (0 min: 5-% B; 100 min: 40-% B; 5 min: 80-% B; 2 min: 100-% B; 13 min: 100-% B). The scan *m/z* range was set to 400–1800. The top 15 most abundant ions with charge states of 2–4, exceeding 20,000 counts were selected for HCD FT MS/MS fragmentation (FTMSMS scans 2–16) and detection in centroid mode.

### Proteomics Data Processing

The nectar and nectary proteome datasets were similarly processed with raw files being created by XCalibur 3.0.63 software and analyzed with Proteome Discoverer (v 1.4.0.228, Thermo Scientific) and were searched against the Uniprot-SwissProt and TrEMBL databases. Nectary dataset search parameters used an MS/MS tolerance of 15 mmu, fixed modification: Methylthio (C), iTRAQ8plex (K), and iTRAQ8plex (N-term), and variable modifications: Oxidation (M), Deamidated (NQ), iTRAQ8plex (Y). The resulting identified proteins underwent statistical validation and filtering using the Scaffold (v 4.6.0 Proteome Software, Inc., Portland, OR, United States) in which the peptide threshold was set to 95% and the minimum number of peptides was set at two. Proteins of non-plant origin were manually removed from datasets. The mass spectrometry proteomics data have been deposited to the ProteomeXchange Consortium^1^ via PRIDE (Vizcaíno et al., 2016) partner repository, with the dataset identifier PXD009810 and 10.6019/PXD009810.

### Statistical Analyses

Relative metabolite concentrations between male and female nectars were compared using a two-tailed independent samples *t*-test with resulting *p*-values corrected for multiple testing using the Benjamini and Hochberg’s method. A Mann Whitney test with Benjamini and Hochberg method for multiple testing correction was used to calculate *p*-values based on the log fold change of protein abundance between male and female nectaries. To visualize proteins with significant differences in abundance between male and female nectaries, adjusted *p*-values were negative log_10_ transformed and plotted against the log_2_ fold difference of protein abundance between male and female in a volcano plot.

Gene Ontology (GO) slimming analysis of nectary proteome annotations was completed using GSEABase (Morgan et al., 2017) with annotations mapped up to the generic GO slim set of terms developed by GO Consortium (The Gene Ontology Consortium, 2000, 2017). GO enrichment analysis of the nectary proteome was implemented using topGO: Enrichment Analysis for Gene Ontology (Alexa and Rahnenfuhrer, 2016) with prior protein-to-GO term mapping completed using the UniProt GO annotation database (Barrell et al., 2009). A Fisher’s exact test was completed to test for enrichment of GO terms using nectary proteins as the background and differentially expressed proteins as the test group.

## Results

### Nectary Morphology

**FIGURE 1:**
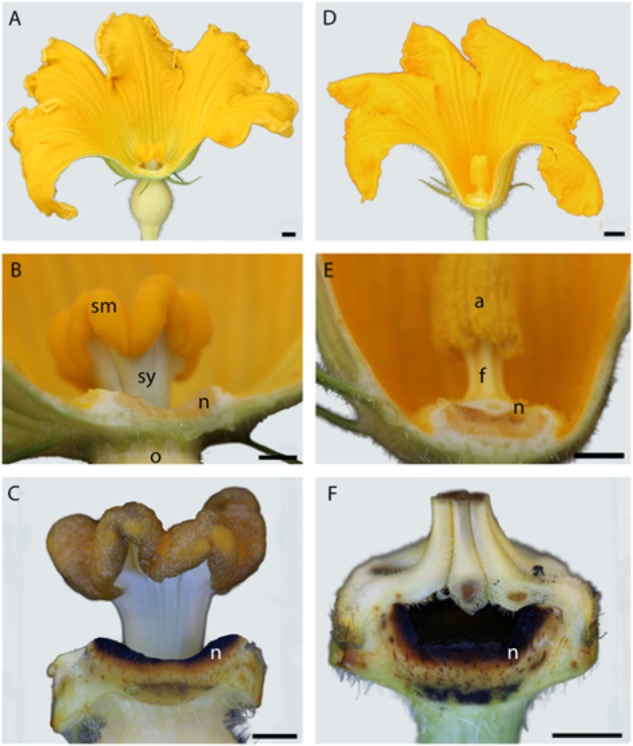
Comparison of female **(A)** and male **(D)** *Cucurbita maxima* flower and nectary morphology. Longitudinal sections of female (left) and male (right) *Cucurbita maxima* flowers. Nectaries of both line the receptacle cavity **(B,E)** and stain black in Lugol potassium iodide solution **(C,F)**. n, nectary; sm, stigma; sy, style; o, ovary; a, anther; f, filament. Scale bars for **A** and **D** = 50 mm; **B, C, E, F** = 5 mm.

In both male and female flowers, the nectary tissue lines the adaxial surface of the receptacle. Morphology and nectary environmental exposure varies by sex. Nectariferous tissue encircles the style column forming a trough for the accumulation of the nectar (**Figures 1A,B**). This nectary position leaves female nectar easily accessible to pollinators. The male nectariferous tissue forms a bowl-like structure below the filaments with the nectar only accessible through slits between pairs of fused filaments (**Figures 1D,E**). Nectaries of both sexes heavily stained black with Lugol indicating that the parenchyma tissue is abundant in amylose-rich starch (**Figures 1C,F**).

### GC-MS Identification of Nectar Metabolites

**FIGURE 2:**
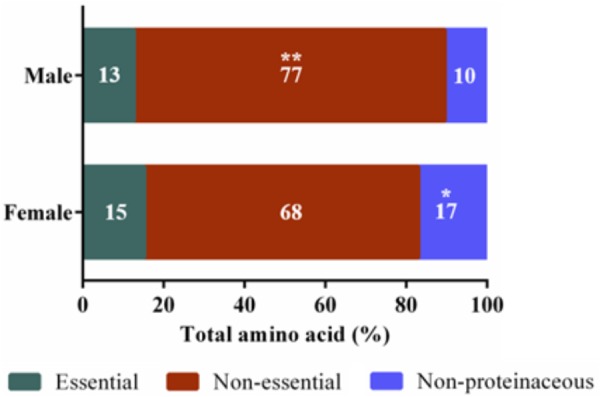
Amino acid categories of *Cucurbita maxima* male and female nectars. Essential amino acids included tryptophan, valine, leucine, isoleucine, threonine, methionine, phenylalanine, and lysine ^∗∗^*p*-value 0.004, ^∗^*p*-value 0.03. *n* = 6, with each replicate consisting of nectar pooled from four flowers.

**FIGURE 3:**
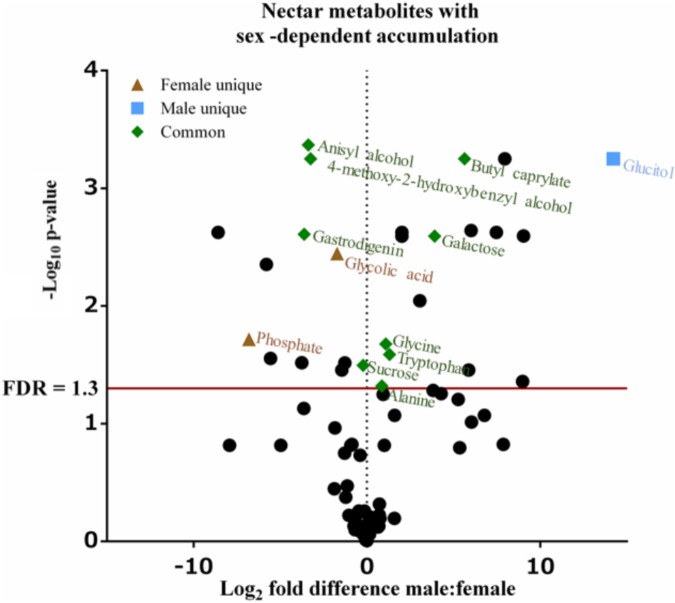
Volcano plot of *Cucurbita maxima* nectar metabolome. Points above the red FDR line represent metabolites with *p*-values <0.05. *n* = 6, with each replicate consisting of nectar from single flowers.

**FIGURE 4:**
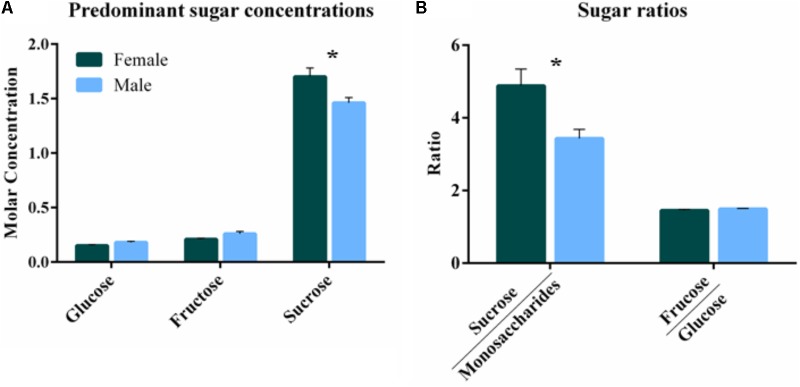
Comparison of *Cucurbita maxima* predominant sugars by flower sex. **(A)** Mean molar concentration ± SE of the predominant sugars. **(B)** Ratios of the disaccharide (sucrose) to the monosaccharides (glucose and fructose) and fructose to glucose for each flower sex. ^∗^*p*-value <0.05. *n* = 6, with each replicate consisting of nectar from single flowers.

Untargeted (GC/GC-MS) and targeted (amino acids) analysis of the nectar metabolome of *C. maxima* led to the detection of 88 analytes, of which 40 could be chemically identified. Classes of identified metabolites from highest to lowest concentrations included sugars, amino acids, sugar alcohols, organic acids, aromatics, esters, and diols. Untargeted metabolite profiling of male and female flowers of *C. maxima* detected a total of 54 analytes (Supplementary Table 1). Targeted profiling of amino acids detected 34 metabolites with 16 identified as proteinaceous amino acids and three as non-proteinaceous amino acids (**Table 1**). Comparison of the molar percentage of these analytes revealed that male nectar contains significantly more non-essential amino acids, and female nectar has a higher proportion of non-proteinaceous amino acids (**Figure 2**). A total of 29 analytes were found to differ significantly in abundance between male and female nectar (**Figure 3**). Of the 29 analytes, 12 were chemically identified, and whereas glucitol was only detected in male nectar, both glycolic acid and phosphate were exclusively detected in female nectar. Regardless of the flower sex, *C. maxima* nectar was sucrose-dominant with a S/[G + F] ratio above 1 (**Figure 4**). Sucrose concentration was significantly greater in female nectars and contributes to a significantly higher S/[G + F] ratio (*p*-value = 0.02, **Figure 4**).

**Table 1:** Amino acids identified in *Cucurbita maxima* nectar reported as mean ± SE (*n* = 6).

Amino Acid	Concentration (μM)		% of total amino acid	
	Female	Male	Female	Male
^∗^Alanine	117 ± 14	212 ± 40	42.8 ± 3.4	52.9 ± 3.4
^∗^Glycine	3.6 ± 0.7	7.6 ± 1.3	1.3 ± 0.2	1.9 ± 0.2
Serine	5.7 ± 1.4	9.5 ± 2.2	1.9 ± 0.4	2.4 ± 0.5
Proline	30.5 ± 4.6	45.2 ± 9.2	11.3 ± 1.6	12.6 ± 3.2
Asparagine	10.2 ± 1.8	7.7 ± 1.6	3.6 ± 0.4	2.1 ± 0.6
Aspartic acid	6.9 ± 2.8	5.2 ± 0.7	2.4 ± 0.8	1.4 ± 0.2
Glutamic acid	11.9 ± 2.4	12.5 ± 1.6	4.4 ± 0.9	3.3 ± 0.5
Tyrosine	0.52 ± 0.15	0.75 ± 0.17	0.17 ± 0.04	0.19 ± 0.04
^∗^Tryptophan	0.23 ± 0.07	0.57 ± 0.11	0.08 ± 0.02	0.16 ± 0.03
Valine	11.9 ± 1.7	14.4 ± 2.5	4.3 ± 0.4	3.6 ± 03
Leucine	3.4 ± 0.4	4.8 ± 1.4	1.2 ± 0.1	1.1 ± 0.2
Isoleucine	11.9 ± 2.0	11.8 ± 2.8	4.4 ± 0.7	2.9 ± 0.6
Threonine	1.3 ± 0.4	2.2 ± 0.3	0.48 ± 0.11	0.59 ± 0.09
Methionine	1.4 ± 0.3	1.9 ± 0.6	0.51 ± 0.10	0.45 ± 0.09
Phenylalanine	11.5 ± 1.8	14.0 ± 1.7	4.2 ± 0.6	3.6 ± 0.3
Lysine	0.24 ± 0.09	0.72 ± 0.31	0.09 ± 0.03	0.19 ± 0.08
β-Alanine	13.1 ± 2.7	15.5 ± 3.1	4.6 ± 0.7	4.4 ± 1.1
GABA	32.9 ± 4.3	21.8 ± 4.4	12.1 ± 1.3	5.9 ± 1.4
4-Hydroxyproline	0.87 ± 0.66	0.54 ± 0.12	0.33 ± 0.24	0.14 ± 0.03

*GABA, γ-aminobutyric acid. ^∗^Indicates metabolites with significantly different concentration between male and female nectar, p-value <0.05.*

### Nectar Proteome

The pooled nectar proteome combined from three individual male and female flowers consists of 45 detected proteins (Supplementary Table 2), 33 of which are present in nectar from both sexes. Two proteins are unique to female nectar and 10 are unique to male nectar. Unique female nectar proteins include galactinol-sucrose galactosyltransferase 2 and cysteine proteinase inhibitor. In the male nectar, eight of the ten unique proteins were characterized as 4-alpha-glucanotransferase, aconitate hydratase, enolase 1, fructose-bisphosphate aldolase, invertase, polygalacturonase, and two different 5-methyltetrahydropteroyltriglutamate-homocysteine methyltransferases. Two unique proteins were uncharacterized proteins from the Uniprot Trembl database. More rigorous sampling in future proteomics analyses may further expand upon these findings, representing the first effort toward cataloging the nectarins of *C. maxima* male and female nectar.

### Nectary Proteome

**FIGURE 5:**
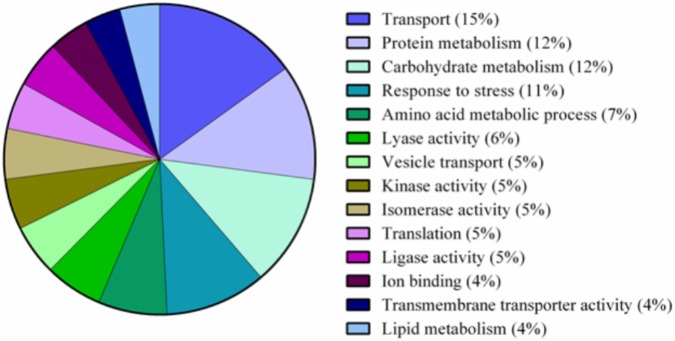
Pie chart of functional classification of proteins found in the nectaries of *Cucurbita maxima.* GO slim categories from the Gene Ontology Consortium were used. Percentages following category name represent the percentage of annotations falling within that category from the top 48% of all GO annotations.

**FIGURE 6:**
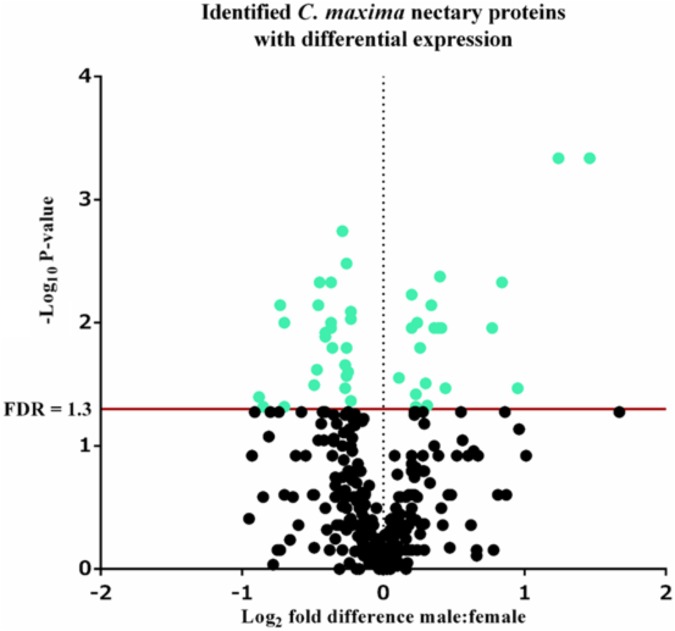
Volcano plot of *Cucurbita maxima* nectary proteome determined by iTRAQ using two female and five male biological replicates with each replicate consisting of the nectary tissue from a single flower. Green points above the red FDR line represent proteins with adjusted *p*-values <0.05.

**FIGURE 7:**
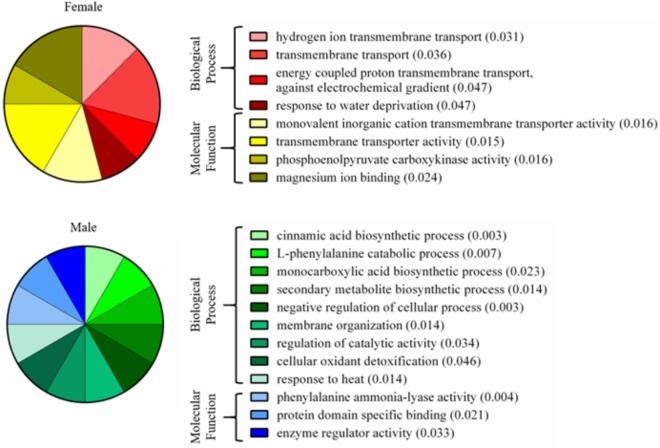
Enriched gene ontology terms of nectary proteins that are differentially expressed between male and female flowers. Pie charts display the most specific enriched GO terms associated with proteins of increased abundance in female or male nectaries. Numbers in parentheses are *p*-values calculated from a Fisher’s exact test for enrichment.

A total of 339 proteins were detected in the nectaries of male and female *C. maxima* flowers using iTRAQ (Supplementary Table 3). To gain a broad overview of functional classifications for the nectary proteome, GO slim analysis was implemented. This revealed a high abundance of proteins related to transport, protein metabolism, carbohydrate metabolism, response to stress, and amino acid metabolic process (**Figure 5**). Statistical comparisons of relative protein abundance revealed that 45 proteins displayed differential expression between male and female nectaries (*p*-value <0.05); 20 of these proteins were more abundant in male nectaries and 25 were more abundant in female nectaries. All 45 proteins have at minimum GO annotation inferred by homology, and descriptive identities are available for 38 of these significant proteins (**Figure 6**). GO enrichment analysis was completed separately for male and female abundant proteins at the three categories of ontology: biological process, molecular function, and cellular component. The most detailed enriched child GO terms for biological process and molecular function are displayed in **Figure 7**. Two cellular component terms, cytosol and cytoplasmic, are female nectary-enriched, while no term is male nectary-enriched. Complete lists of input GO IDs and enriched terms are listed in Supplementary Tables 4 and 5, respectively. Female nectary-enriched GO terms relate to transmembrane transport of ions, magnesium ion binding, response to water deprivation, and carboxy-lyase catalytic activity. Most male nectary-enriched GO terms are related to phenylalanine ammonia-lyase, an enzyme involved in phenylpropanoid biosynthesis (**Figure 7**). Additional enriched GO terms include cellular oxidant detoxification, negative regulation of cellular process, response to heat, and membrane organization.

## Discussion

The synthesis and secretion of nectar is a highly dynamic process, which is only recently beginning to be understood through the robustness of “omics” technologies. Presently, there are two competing models of nectar secretion supported by ultrastructural analyses or molecular genetic studies. In the first model, merocrine (granulocrine), pre-nectar metabolites are transported symplastically through plasmodesmata until they reach cells near the nectary surface, where they are packed into ER or Golgi body vesicles for later fusion with the plasma membrane and secretion. The second model, eccrine, depends on plasma membrane localized pores and transporters instead of vesicles for exporting nectar metabolites from the nectary cells (Roy et al., 2017). This model is supported by the conservation of SWEET9, a plasma membrane sucrose uniporter, within mature nectaries of Brassicaceae and Solanaceae (Lin et al., 2014). Once nectar is secreted, it is far from a complex static solution of primarily sugars. Rather, nectar is in a dynamic equilibrium, responsive to environmental conditions and can undergo post-secretory modifications via the action of catalytic nectarins which act on carbohydrates or generate anti-microbial agents such as hydrogen peroxide (Carter and Thornburg, 2004; González-Teuber et al., 2010; Nepi et al., 2011a,b). The primary objective of the current study was to examine potential sex-dependent variation in *C. maxima* nectar composition at the level of the metabolome and proteome extending existing knowledge of biologically relevant sex-dependent nectar variation with regards to nectar composition and rates of nectar production (Nepi et al., 2001; Ashworth and Galetto, 2002). Secondarily, this study aimed to propose metabolic links between nectar metabolites and proteins present in the nectary and nectar proteomes.

### Nectar Metabolomics

Compared to the nectar of male flowers, female nectar of *C. maxima* has significantly more sucrose and a higher sucrose to hexose ratio. These findings contrast with previous studies of *C. pepo* and *C. maxima* (Nepi et al., 2001; Ashworth and Galetto, 2002) that found little difference in abundances of the three predominate sugars between male and female nectars. This variation in the findings between the studies may be due to differences in environmental growing conditions of the plants as well as variation in species and cultivar. This may be particularly significant in light of the fact that these sugars influence defining characteristics of nectar, such as viscosity and its ability to attract pollinators (Baker and Baker, 1983). A second sugar, galactose, present at much lower concentrations than sucrose, glucose, and fructose, was significantly less abundant in female nectar. Because bees can easily judge sugar composition and nectar volume (Hendriksma et al., 2014), the variation in both sucrose and galactose content observed in *C. maxima* nectars may influence the degree to which bees are more attracted to female flowers (Ashworth and Galetto, 2002).

Amino acids are the second most common class of metabolites that occur in nectar, but their concentrations are 100 to 1,000 times less than the predominant sugars (Roy et al., 2017). In the present study, 16 proteinaceous amino acids and three non-proteinaceous amino acids were identified in both male and female nectar of *C. maxima*. Over 70% by mole of the identified amino acids were accounted by alanine, proline, GABA, and β-alanine. Although this is similar to the nectar of *C. pepo* (Nepi et al., 2012), there is a striking difference in the relative proportion of proline and alanine; in *C. pepo* proline is the most abundant amino acid followed by alanine (30% and 5% respectively) (Nepi et al., 2012), in *C. maxima* nectar, their relative order is reversed, with alanine being the most abundant amino acid (40%), followed by proline (11%). Proline often occurs as an abundant nectar amino acid, and has multiple effects on bees, including providing a desirable flavor and serving as a muscle stimulant giving a quick burst of energy for flight take-off (Carter et al., 2006; Teulier et al., 2016). The finding of two relatively high abundant non-proteinaceous amino acids, GABA and β-alanine, in *C. maxima* nectars agrees with commonly observed amino acid profiles of floral nectars (Nepi et al., 2012). They are both thought to promote insect flight, while GABA is also implicated as an antimicrobial agent used by plants in response to wounding (Chevrot et al., 2006). Since GABA is also a neurotransmitter (Nepi, 2014), it is possible that it may directly influence bee behavior.

The most significant differences between male and female nectars, in regard to amino acids, was the relative abundance of tryptophan, alanine, and glycine, which were specifically more concentrated in male nectar. These amino acids appear to alter bee feeding preferences, with tryptophan and alanine functioning as bee attractants, while glycine is a deterrent (Bertazzini et al., 2010; Hendriksma et al., 2014). Based on these previous studies, it is unclear whether the statistically significant variation in tryptophan, alanine, and glycine would influence bee feeding preferences between male and female flowers. Studies are needed to determine the biologically relevant ratio of the attractants (alanine and tryptophan) to deterrents (glycine) needed to alter bee preferences as mixtures of amino acids can have synergistic effects on bee preferences. When the proportions of essential, non-essential, and non-proteinaceous amino acids are compared by sex, we found that the male nectar has a significantly higher proportion of non-essential amino acids, largely due to increased concentrations of alanine and glycine. Female nectar contained more non-proteinaceous amino acids, specifically GABA (*p*-value = 0.009) which as previously stated may confer anti-microbial properties important in keeping the gynoecium free of pathogenic infection.

In addition to sugars and amino acids, nectar often contains a diversity of primary and secondary metabolites whose functions are wide ranging and include pollinator rewards, preservatives, and defense against pathogens (Stevenson et al., 2017). In our study, additional primary metabolites (glucitol, glycolic acid, and phosphate) and secondary metabolites (4-methoxy-2-hydroxybenzyl alcohol, anisyl alcohol, butyl caprylate, and gastrodigenin) displayed sex-dependent difference in accumulation. To our knowledge, no nectar-specific functions are reported for these metabolites, although the sex-dependent accumulation of these metabolites may indicate that they influence pollinator attraction to male and female flowers. Specifically, glucitol was only detected in male nectar, whereas glycolic acid and phosphate were restricted to female nectar. Butyl caprylate, a fragrant ester, which was more abundant in male nectar, has previously been detected in floral volatile profiles of orchids (Kaiser, 1993). In female *C. maxima* nectar, 4-methoxy-2-hydroxybenzyl alcohol, anisyl alcohol, and gastrodigenin are present at higher concentrations as compared to male nectars. Anisyl alcohol, similar to butyl caprylate, is not only a floral scent present in orchids (Kaiser, 1993) but also occurs in anise, honey, and vanilla (Scognamiglio et al., 2012). Gastrodigenin, also known as 4-hydroxybenzyl alcohol, is a known antioxidant occurring in a variety of plants (Lim et al., 2007).

### Nectar Proteome

Prior characterization of nectarins have indicated that these proteins function as either anti-microbials or as enzymes that alter nectar carbohydrate chemistries. Consistent with the latter observation, 9 of the 10 proteins that are unique to male nectar are enzymes that act on carbohydrates, the exceptions being 5-methyltetrahydropteroyltriglutamate–homocysteine methyltransferase. These carbohydrate-modifying enzymes include invertase, which catalyzes the hydrolysis of sucrose to glucose and fructose. Invertases have previously been reported in other nectars and studied extensively in *Acacia* extrafloral nectar and *C. pepo* floral nectar (Heil et al., 2005; Nepi et al., 2012). Six of the characterized male unique proteins (4-alpha-glucanotransferase, 5-methyltetrahydropteroyltriglutamate–homocysteine methyltransferase, aconitate hydratase, enolase 1, fructose-bisphosphate aldolase, and polygalacturonase) have not previously been reported in nectar, but annotation data indicate that they are either located in cytoplasm of cells or extracellular space, supporting their detection in *C. maxima* nectar.

Female nectar contains two unique nectarins, a cysteine proteinase inhibitor and galactinol–sucrose galactosyltransferase 2. The first of these has previously been reported in the floral nectar proteome of *Liriodendron tulipifera* (Zhou et al., 2016), but the latter has not been reported in nectars. The galactosyltransferase has the potential to modify the carbohydrate profile of female nectar as it functions in galactose metabolism, generating myo-inositol and raffinose from galactinol and sucrose.

In addition to the sex-specific nectarins, 33 other proteins were detected in the nectar proteome of both *C. maxima* flower sexes. Several of these were previously reported in nectars of other species, including malate dehydrogenase in petunia nectar (Hillwig et al., 2011), β-glucosidase in nectar of *Acacia hindsii* and *A. collinsii* EFN (González-Teuber et al., 2010), α-galactosidase in common tobacco nectar (Zha et al., 2012), and glutathione *S*-transferase and a heat shock protein both of which occur in the nectar of *Liriodendron tulipifera* (Zhou et al., 2016). A second group of nectarins (i.e., adenosylhomocysteinase 1, β-galactosidase, and α-glucan phosphorylase) were identified in both male and female *C. maxima* nectars, but they had not previously been reported in nectars of other species. These proteins were also undetectable in the nectary proteome of *C. maxima* flowers. The absence of these proteins in the proteome of the nectary, where they are synthesized, may indicate that these proteins are efficiently and rapidly secreted into the nectar. It is also possible that the complexity of the nectary proteome masks the identification of nectar proteins at their site of synthesis.

### Nectary Proteome

The major functional classifications of the *C. maxima* nectary proteome includes proteins involved in transport, protein metabolism, carbohydrate metabolism, response to stress, and amino acid metabolism (**Figure 4**), and these are similar to those found in *Acacia cornigera* (Orona-Tamayo et al., 2013) and *Ricinus communis* (Shah et al., 2016) extrafloral nectary proteomes. These functional classifications are expected as carbohydrates and amino acids are the most abundant nectar metabolites and require extensive transport within the nectary. GO enrichment analysis of nectary proteins with increased female abundances indicate that female-enriched GO terms are associated with proteins functioning as plasma membrane proton pumps and central metabolism, specifically gluconeogenesis, glycolysis, lipid metabolism, and the citric acid cycle. Proteins associated with male nectary-enriched GO terms were related to cinnamic acid biosynthesis and neutralization of superoxide radicals and hydrogen peroxide. If pumpkin nectaries generate high levels of reactive oxygen species (ROS), like tobacco (Carter and Thornburg, 2004; Carter et al., 2007), it would not be surprising if they also contain mechanisms to mitigate their potentially damaging reagent.

As a whole, the nectary proteome in conjunction with previous cucurbit nectary literature supports an eccrine model of nectar secretion where plasma membrane (PM) H-^+^-ATPase provides the energy for active transport of solutes into the apoplasm of *C. maxima* nectaries. In the current study, functional classification of nectary proteins and GO term enrichment analyses both revealed an abundance of ATPase transmembrane transporters specific for hydrogen ions, indicating the important role of PM-H-^+^-ATPase in active *C. maxima* nectaries. This finding agrees with the pressure-driven mass flow model of nectar movement from parenchyma tissue into the apoplast, in which PM-H-^+^-ATPase provides energy for active transport of solutes into the apoplast creating an osmotic gradient for the movement of water through aquaporins. The resulting hydrostatic pressure in the apoplast produces mass flow of nectar out of the nectary tissue and to the surface (Vassilyev, 2010). Additionally, it has also been suggested that nectar secretion in *Cucumis sativus* requires PM-H-^+^-ATPase, as ATPase-specific activity peaks at anthesis (Peng et al., 2004).

Previous ultrastructural analyses of *C. pepo* demonstrate that the nectary cells are devoid of extensive ER and Golgi making the vesicle dependent merocrine model unfavorable when compared to the eccrine model (Nepi et al., 1996). While the eccrine model may predominate, merocrine is still needed for vesicular-based transport of nectarins, and may be important in *C. maxima* nectaries as vesicle transport is frequency functional classification of its proteome (**Figure 4**) (Roy et al., 2017). The eccrine model of nectar synthesis and secretion that is supported by molecular evidence from Brassicaceae and Solanaceae expresses four metabolic processes: (1) starch degradation, (2) sucrose synthesis, (3) export of sucrose into apoplasm via SWEET9, and (4) extracellular hydrolysis of sucrose via CELL WALL INVERTASE4 (CWINV4) (Ruhlmann et al., 2010; Lin et al., 2014; Thomas et al., 2017). The *C. maxima* nectary proteome determined herein supports the occurrence of the first two of these processes, as both a β-amylase for starch hydrolysis and sucrose-phosphate synthase that function in sucrose biosynthesis are present. Homologs of SWEET9 and CWINV4 were not identified within the nectary proteome under the specified data filtering conditions. Moreover, as a transmembrane protein, SWEET9 may not have been extracted from the nectary tissue as the methodology was not ideal for extraction of membrane proteins. CWINV4 may not be highly expressed in *C. maxima* nectaries which produce a sucrose dominant nectar as compared to the hexose dominant nectar produced by the Arabidopsis nectaries; the expression of CWINV4 is essential for functional development of nectaries in Arabidopsis (Ruhlmann et al., 2010).

### Metabolic Links Between Nectar Metabolites and Proteomes

Nectarins commonly alter nectar carbohydrates. In our datasets, significant differences in carbohydrate abundance, specifically galactose and sucrose, may be explained by the unique presence of galactinol–sucrose galactosyltransferase 2 and invertase in the nectar of female and male flowers respectively. Galactose is significantly less in female nectar which also contains galactinol–sucrose galactosyltransferase 2 which is not found in male nectar. This enzyme utilizes galactose as a substrate, leading to the production of myo-inositol and raffinose, a primary transport sugar in cucurbits (Zhang et al., 2010); this may explain why galactose levels are lower in female nectar as compared to male. A second potential example of post-secretory carbohydrate alterations is suggested by the slight but statistically significant reduction in sucrose content of male nectar which contains an invertase that is not detectable in female nectar. Invertases catalyze the hydrolysis of sucrose to glucose and fructose. The difference in sucrose concentration between male and female nectar may only be slight due to the ability of the male nectary to maintain a nectar equilibrium. In *C. pepo* for example, male flowers can regulate water and sugar content to maintain nectar homoeostasis during secretion (Nepi et al., 2011b). This ability to regulate sugar content may nullify the impact of invertase within the male nectar of *C. maxima*.

## Conclusion

In this study, we demonstrated an existence of sex-dependent variation in male and female floral nectaries and nectar of *C. maxima* as determined by proteomics and metabolomics. Nectar metabolites that varied in composition range from carbohydrates, amino acids, and specialized metabolites, and the nectarin profiles. Nectarins specific to a single nectar sex were linked to observed differences in the nectar metabolomes. Additionally, the nectary proteome supported aspects of the eccrine model of nectar secretion and pressure-driven mass flow utilizing PM-H-^+^-ATPase.

## Author Contributions

CJC conceived and planned the research. EC completed sample collection and metabolomics analyses. Proteomics experimental work and analyses were completed by ME, DS, EC, and PvA. Manuscript was written by EC, PvA, and BN.

## Conflict of Interest Statement

CJC and BN are the co-PIs on the National Science Foundation grant that supported this study. The remaining authors declare that the research was conducted in the absence of any commercial or financial relationships that could be construed as a potential conflict of interest.

## Footnotes

1 http://www.proteomexchange.org/
